# 

**Published:** 1861-05

**Authors:** 


### RECEIPTS NOT HERETOFORE ACKNOWLEDGED.

S. M. Reynolds, Ill., vols. 2, 3 and half 4, $5; J. J. Kackley, Iowa, vol. 4, $2; P. A. Allaire, Ill., vols 3 and 4, $4; T. B. Tuttle, Iowa, vol 3, $2; F. W. Floyd, Ill., vol. 3, $2; Robt. W. Earll, Wis., vol. 4, $2; E. G. Dyer, Wis., vol. 4, $2; Mord. Davis, Ill., vols. 2 and half 3, $3; J. W. Green, Ind., vols. half 2, 3 and 4, $5; John Wright, Ill., vol. 3, $2. Omitted last month: John Rea, Iowa, vol 4, $2; J. C. Mills, Wis., vols. half 3 and 4, $3; H. A. Youmans, Wis., vol. 4, $2.

CORRECTIONS, &c., received and noted on books, from Drs. J. N. Denny, Lucius Clark, John Allen, C. D. Henton, D. B, Wren, S. E. Robinson, C. A. Logan, Henry Folke, A. DeArmond, A. A. & H. C. Rawson, M. Wiley.

